# Supplementary material for: Active Manipulation of the Spin and Orbital Angular Momentums in a Terahertz Graphene-Based Hybrid Plasmonic Waveguide
Source: Nanomaterials (Basel). 2020 Dec 5;10(12):2436. doi: 10.3390/nano10122436 (PMC7762202; doi:10.3390/nano10122436)
Supplement: Supplementary file 1 [file nanomaterials-10-02436-s001.pdf]

Supplementary Materials:

Active Manipulation of The Spin and Orbital Angular Momentums in a Terahertz Graphene-Based Hybrid Plasmonic Waveguide

Ziang Wang [1], Qilong Tan [2], Yong Liang [1], Xia Zhou [1], Wen Zhou [3,*] and Xuguang Huang [1,*]

[1] School of Information and Optoelectronic Science and Engineering, South China Normal University, Guangzhou 510006, China; 2018022060@m.scnu.edu.cn (Z.W.); 2018022045@m.scnu.edu.cn (Y.L.); XZhou@m.scnu.edu.cn (X.Z.)

[2] School of Physics and Telecommunications Engineering, South China Normal University, Guangzhou 510006, China; tanqilong@m.scnu.edu.cn

[3] Department of Materials, University of Oxford, Oxford OX1 3PH, UK

* Correspondence: wzhoucuhk@gmail.com (W.Z.); huangxg@scnu.edu.cn (X.H.)

The detailed fabrication scheme for the hybrid waveguide as shown in Figure S1 could be as follows: Polysilicon is deposited on SiO_2 substrate by Low Pressure Chemical Vapor Deposition (LPCVD); (2) SiO_2 is deposited on Polysilicon by Plasma Enhanced Chemical Vapor Deposition (PECVD) at 300 °C; (3) 5 graphene layers grown by Chemical Vapour Deposition (CVD) were wet transferred onto the surface of SiO_2 layer; (4) thin film Al_2O_3 is deposited on graphene layer by Atomic Layer Deposition (ALD) as a capping layer for graphene; (5) SiO_2 is deposited on Al_2O_3 film by PECVD at 300 °C; (6) GaAs grown on SiO_2 layer by CVD; (7) Using reactive ion Etching (RIE) to etch GaAs to form the GaAs core; (8) SiO_2 is deposited around GaAs core by PECVD at 300 °C and Chemical-mechanical polishing of SiO_2 to create a flat top; (9) Using RIE to etch SiO_2 to form the SiO_2 core; (10) polysilicon is deposited on the side of SiO_2; (11) SiO2 is deposited on the side of polysilicon by PECVD; (12) Wet transfer of CVD graphene,UV photolithography opening windows for photoresist on graphene, O2 plasma etching to create sidewall graphene; (13) two times UV photolithography opening windows, left side photoresist window for HF wet etching of SiO_2 and Al_2O_3 down to graphene, right side photoresist window for RIE etching down to polysilicon (which is an etching stop layer, in the meanwhile graphene will be burned away by plasma); (14) use UV photolithography to open windows for e-beam evaporating Au layers, and perform lift off to form electrodes.

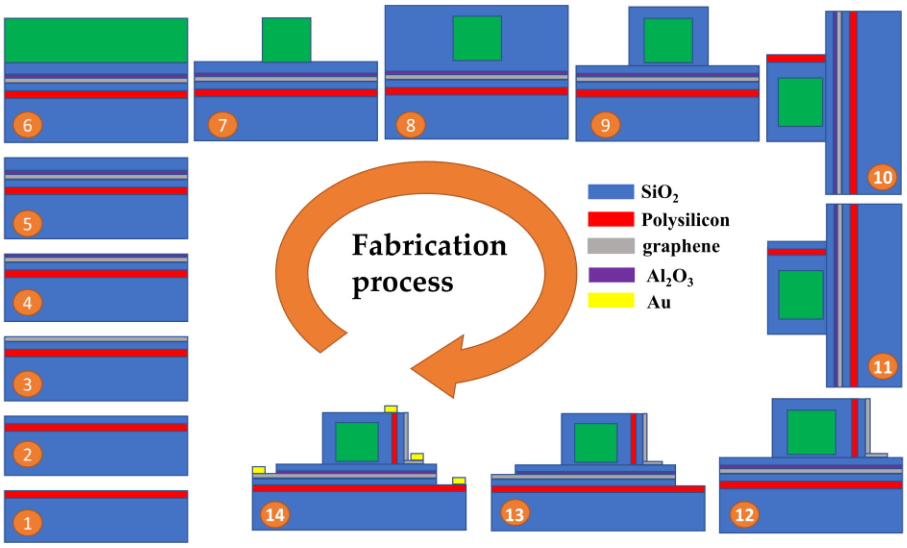

Figure S1. Fabrication process of the proposed graphene-based hybrid plasmonic waveguide.
